# Optimization of whole-cell biotransformation for scale-up production of α-arbutin from hydroquinone by the use of recombinant *Escherichia coli*

**DOI:** 10.1186/s13568-019-0820-7

**Published:** 2019-06-28

**Authors:** Linjiang Zhu, Min Xu, Changxin Lu, Luyi Chen, Anjie Xu, Jingyi Fang, Hanchi Chen, Yuele Lu, Yongxian Fan, Xiaolong Chen

**Affiliations:** 1 Institute of Fermentation Engineering, Zhejiang University of Technology, 18 Chaowang Road, Hangzhou, 310014 China; 2 College of Biotechnology and Bioengineering, Zhejiang University of Technology, Hangzhou, 310014 China; 3 College of Chemical Engineering, Zhejiang University of Technology, Hangzhou, 310014 China

**Keywords:** α-Arbutin, Whole-cell catalysis, Scale-up, Glycosylation, Amylosucrase

## Abstract

α-Arbutin is an effective skin-whitening cosmetic ingredient and hyperpigmentation therapy agent. It can be synthesized by one-step enzymatic glycosylation of hydroquinone (HQ), but limited by the low yield. Amylosucrase (Amy-1) from *Xanthomonas campestris* pv. *campestris* 8004 was recently identified with high HQ glycosylation activity. In this study, whole-cell transformation by Amy-1 was optimized and process scale-up was evaluated in 5000-L reactor. In comparison with purified Amy-1, whole-cell catalyst of recombinant *E. coli* displays better tolerance against inhibitors (oxidized products of HQ) and requires lower molar ratio of sucrose and HQ to reach high conversion rate (> 99%). Excess accumulation of glucose (0.6–1.0 M) derived from sucrose hydrolysis inhibits HQ glycosylation rate by 46–60%, which suggests the importance of balancing HQ glycosylation rate and sucrose hydrolysis rate by adjusting the activity of whole-cell catalyst and HQ-fed rate. Using optimal conditions, 540 mM of final concentration and 95% of molar conversion rate were obtained within 13–18 h in laboratory scale. For industrial scale-up production, 398 mM and 375 mM of final concentration with high conversion rates (~ 95%) were obtained in 3500-L and 4000-L of reaction volume, respectively. These yields and productivities (4.5–4.9 kg kL^−1^ h^−1^) were the highest by comparing to the best we known. Hence, high-yield production of α-arbutin by batch-feeding whole-cell biotransformation was successfully achieved in the 5000-L reaction scale.

## Introduction

Arbutin, as a natural hydroquinone (HQ) glucopyranoside derivative, is an effective and safe hyperpigmentation therapy agent and skin-whitening cosmetic ingredient (Desmedt et al. 2016). It strongly inhibits tyrosinase activity and reduces the formation of melanin without adverse side effects like irritation, allergic contact dermatitis, and other cytotoxicity. Natural arbutin, with β-anomeric glycoside bond (β-arbutin), can be isolated from berry-producing plants such as blueberry, cranberry, marjoram, and most pear species (Lukas et al. 2010; Migas and Krauze-Baranowska 2015). The other arbutin isomer is α-arbutin with α-anomeric glycoside bond. Both of isomers are commonly used in skin-whitening cosmetics and hyperpigmentation therapy (Migas and Krauze-Baranowska 2015), while α-arbutin displays ten times stronger inhibitory activity than β-arbutin (Sugimoto et al. 2004). With the rapid growth of global skin-whitening cosmetics market, the environment-friendly production of α-arbutin attracts wide interests since it can be synthesized by one-step enzymatic glycosylation of HQ.

**Fig. 1:**
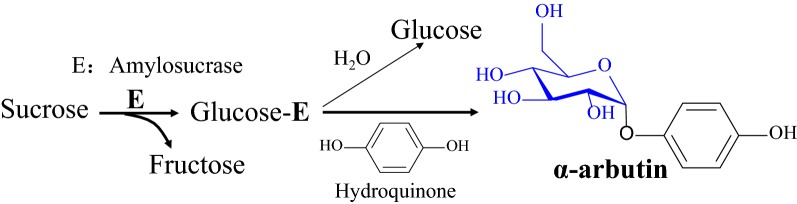
Enzymatic glycosylation of hydroquinone by amylosucrase from *X. campestris* sp. *campestris* 8004 for synthesis of α-arbutin. Amylosucrase (Amy-1) used sucrose as glycoside donor to glycosylate hydroquinone. Amy-1 also hydrolyzed sucrose to produce fructose and glucose

β-Arbutin used to dominant the arbutin material market due to its lower production cost though chemical synthesis than α-arbutin. However, the drawbacks of chemical synthesis are obvious such as labor-intensive activation, protection procedures, low overall yields and a large amount of waste (Desmet et al. 2012). α-Arbutin has been reported to be synthesized by environment-friendly one-step enzymatic transformation since 1990s (Seo et al. 2012; Zhu et al. 2018), but industrial production was limited by the low yield. α-Amylase was firstly reported with HQ glycosylation activity for α-arbutin production but with a low conversion rate of 32%, besides, the products were the mixture of α-arbutin and hydroquinone oligoglucosides (Nishimura et al. 1994). It was later reported that α-amylase could obtain a high concentration (> 100 g/L of α-arbutin), displaying a great potential for industrial production (Kazuhisha et al. 2007). Subsequently, sucrose phosphorylase from *Leuconostoc mesenteroides* and α-glucosidase from *Xanthomonas campestris* were reported with a higher molar conversion rate of 60% and 93%, respectively, but only a low concentration of α-arbutin (~ 10 g/L) was obtained (Kitao and Sekine 1994; Kurosu et al. 2002; Sato et al. 2012). In recent years, various enzymes were newly identified with HQ glycosylation activity including dextransucrase from *L. mesenteroides*, cyclodextrin glucanotransferase from *Thermoanaerobacter* sp., amylosucrases from *Cellulomonas carboniz*, *Deinococcus geothermalis*, and *X. campestris* (Mathew and Adlercreutz 2013; Seo et al. 2009; Yang et al. 2019; Yu et al. 2018). Amylosucrase displayed a highest HQ glycosylation activity among these enzymes, for example, obtaining > 90% of molar conversion rate and 60 g/L of concentration (Yang et al. 2019). We also recently identified the amylosucrase (Amy-1) from *Xanthomonas campestris* with > 95% molar conversion rate and > 80 g/L of α-arbutin concentration (Fig. 1) (Zhu et al. 2019). Therefore, remarkable progress in biosynthesis of α-arbutin has been made in recent years.

Whole-cell catalysis by recombinant *E. coli* cells was proposed to be better way for α-arbutin synthesis by Amy-1 due to the potential better tolerance against the oxidized products of HQ (Zhu et al. 2019). Besides, whole-cell catalyst is intrinsically the cheapest and easy-to-scale-up catalyst as it circumvents the need of cell lysis and enzyme purification (Wachtmeister and Rother 2016). Therefore, to achieve industrial scale production of α-arbutin, whole-cell catalysis was optimized and scale-up production in 5000 L reactor was evaluated in this study.

## Materials and methods

### Strain and culture conditions

The recombinant *Escherichia coli* BL21 (pET28a-amy1) was used in this study, which overexpressed amylosucrase (NCBI-Protein ID: AAY47880) from *X. campestris* sp. *campestris* 8004 (Zhu et al. 2019). It was grown in the LB (Luria–Bertani) medium supplemented with 50 μg/mL of kanamycin at 37 °C, 200 rpm. Fermentation medium contained yeast extract 15 g/L, tryptone 20 g/L, glycerol 10 g/L, KH_2_PO_4_ 0.17 M, K_2_HPO_4_ 0.72 M, MgSO_4_·7H_2_O 0.5 g/L. Glycerol feeding solution contained glycerol 300 g/L; MgSO_4_·7H_2_O 10 g/L, vitamin B1 24 mg/L, trace salt solution 5 mL/L. Trace salt solution contained CoCl_2_·6H_2_O 2.5 g/L, MnCl_2_·4H_2_O 15 g/L, CuCl_2_·2H_2_O 1.5 g/L, boric acid 3 g/L, Na_2_MoO_4_·2H_2_O 2.5 g/L, Zn (CH_3_COO)_2_·2H_2_O 13 g/L, ferric citrate 12.5 g/L.

### Fermentation and preparation of whole-cell catalyst

A seed culture was prepared in a 500 mL flask containing 50 mL of LB medium supplemented with 50 μg/mL of kanamycin at 37 °C, 220 rpm. Fed-batch cultures were grown in a 2.5 L fermenter (Bioflo 110; New Brunswick Scientific Co., Shanghai, China) containing 1.0 L of initial fermentation medium. The initial culture conditions were 30 °C, 2.5 L/min of air and agitation at 400 rpm. The pH was maintained at 6.8 by adding 28% (v/v) ammonia hydroxide. The dissolved oxygen concentration was kept at > 25% of air saturation by automatically coupling the agitation speed (400–960 rpm). When an optical density at 600 nm (OD_600_) of cultures reached ~ 15, culture conditions were shifted to 24 °C and 5.0 L/min of air. 400 mL of glycerol feeding solution started to be added with constant speed (12 mL/h/L of initial fermentation volume). 150 mL of lactose solution (20% of lactose) also started to be added by a speed of 36 mL/h/L of initial fermentation volume. When OD_600_ of cultures reached to ~ 70, additional 150 mL of lactose solution was added with the same speed. After ~ 40 h, when agitation speed decreased to < 500 rpm, the fed-batch fermentation was ended and OD_600_ of culture reached to > 100. The obtained cultures were stored at 4 °C for 2 weeks until they were used.

For the preparation of whole-cell catalysis, the culture was centrifuged by 5000×*g* at 4 °C for 10 min and the cells were washed and resuspended using 50 mM phosphate buffer system (PBS) (pH 7.0). The obtained cell suspension was used for glycosylation activity assay, and then diluted to different cell density as whole-cell catalyst.

### Glycosylation activity assay of whole-cell catalysis

The glycosylation activity assay of whole-cell catalysis was performed in 100 mL round-bottom flask with 50 mL reaction volume, which was incubated in a thermostatic waterbaths with a magnetic stirrer at 30 °C for 10 min. The reaction mixtures contained 1.2 M of sucrose, 30 mM of HQ, 5 OD_600_ of cells, 50 mM PBS (pH 7.0). Each experiment was carried out in triplicate. One unit of glucoside transfer activity was defined as the amount of whole-cell catalyst that produced 1 mM of α-arbutin per minute in above given conditions.

The amounts of HQ and α-arbutin were measured by HPLC using a Shimadzu LC-20A system with SPD-MzoA Diode Array Detector (Kyoto, Japan) as described previously (Zhu et al. 2019).

### Optimizing catalytic reaction conditions for whole-cell biotransformation

Glycosylation reaction by whole-cell catalyst was routinely performed in 100 mL round-bottom flask with 50 mL reaction volume, which was incubated in a thermostatic waterbaths with a magnetic stirrer. Different pH buffers in the range of 4.0–10.0 were compared, including 50 mM of sodium acetate buffer (pH 3.9–6.5), 50 mM of PBS (pH 5.6–7.8), and 10 mM of H_3_BO_3_-NaOH-KCl buffer (pH 7.6–10.0). The reaction mixtures all contained 5 OD_600_ of cells (0.4 U/mL), 1.2 M of sucrose, and 50 mM of hydroquinone. The glycosylation activities of whole-cell catalyst at various temperatures ranging from 15 to 60 °C were also evaluated and the molar conversion rates in 15 min were compared. The thermostability at different temperature was compared. The cell suspension solutions were incubated in thermostatic waterbaths with different temperature ranging from 20 to 45 °C. After incubated for 0.5–2.0 h, cell suspension solutions were taken for glycosylation activity assay. Each experiment mentioned above was carried out in triple.

### Evaluating inhibitory effect of substrates, products and *p*-benzoquinone on activity of whole-cell catalyst

The inhibitory effects of HQ were evaluated with different initial HQ concentration at 20, 40, 50, 80, 100 and 120 mM in the reaction mixtures containing 5 OD_600_ of cell (0.4 U/mL) or 0.4 U/mL of Amy-1, and 1.2 M of sucrose in 50 mM PBS (pH 7.0). The amount of α-arbutin and HQ were monitored by HPLC analysis.

Product inhibition of glucose and fructose was tested. Various initial concentrations including 0 M (control), 0.1 M, 0.2 M, 0.4 M, 0.6 M, 0.8 M, and 1.0 M were used. The mixtures contained 5 OD_600_ of cell (0.4 U/mL), 50 mM of HQ, 1.0 M of sucrose in 50 mM of PBS (pH7.0). After 0.5 h of reaction, the molar conversion rates were calculated as following: (C_0.5Arb_/(C_0.5Arb_ + C_0.5HQ_))*100, where C_0.5Arb_ is molar concentration of arbutin (mM) at 0.5 h; C_0.5HQ_ is the HQ molar concentration (mM) at 0.5 h. Relative activity was calculated by comparison with the molar conversion rate of control.

Different molar ratios (1:1, 5:1, 10:1, 20:1 and 40:1) of glucoside donor sucrose and acceptor hydroquinone were prepared by varying the sucrose concentration (30 mM–1.2 M) with constant HQ concentration (30 mM). The reactions were catalyzed by 10 OD_600_ of cell (0.8 U/mL) or 0.8 U/mL of Amy-1 at 30 °C.

The inhibitory effect of *p*-benzoquinone was evaluated in the reaction mixture containing 20 OD_600_ of cell (1.6 U/mL) or 1.6 U/mL of Amy-1, 30 mM of HQ, 1.2 M of sucrose, and different initial concentration of *p*-benzoquinone including 0, 0.1, 0.5, 1.0 and 5.0 mM, respectively. The mixtures were incubated at 30 °C. Each experiment mentioned above was carried out in triple.

### Sucrose hydrolysis activity assay

The sucrose hydrolysis activity of whole-cell catalysis was performed in 100 mL round-bottom flask as mentioned above. The reaction mixtures contained 1.2 M of sucrose, 5 and 10 OD_600_ of cells, 50 mM PBS (pH 7.0). The mixtures were incubated for 19 h at 30 °C. The amounts of sucrose, glucose and fructose were measured by HPLC using an Agilent 1260 Infinity system with evaporative light scattering detector G4260B ELSD (Agilent, USA). 0.1 mL of the reaction mixture was added into 0.9 mL of deionized water. The sample was heated for 5 min at 100 °C, and then centrifuged (15,000×*g*, 5 min). The supernatant was further diluted by 20-fold and filtrated with 0.2 nm PTFE membrane for HLPC analysis. HPLC conditions: column, Xtimate Sugar-Ca (300 mm × 7.8 mm, 5 μm) (Welch Materials Inc., Shanghai, China); mobile phase, ultrapure water; flow rate, 0.5 mL/min; and temperature, 70 °C; injection volume, 10 μL. The parameters for the ELSD were as follows: evaporator temperature: 90 °C, nebulizer temperature: 50 °C, gas flow: 1.2 SLM, data rate: 40 Hz, LED intensity: 100%, PMT gain: 1, smooth: 10. Each experiment was carried out in triplicate.

### Batch-feeding whole-cell catalysis in laboratory scale

Batch-feeding whole-cell catalysis was performed in 100 mL round-bottom flask with 50 mL reaction volume, which was incubated in a thermostatic waterbaths with a magnetic stirrer at 30 °C. The initial reaction mixture contained 1.2 M sucrose, 60 mM HQ, and different concentrations of cells (OD_600_ = 1.0, 5.0, 10, 20) in 50 mM PBS (pH 7.0). Every 10–30 min, 0.1 mL of samples was added into 0.9 mL of stop solution, and then used for HPLC analysis of HQ and α-arbutin. After that, different amounts of solid HQ were added into the reaction mixtures to supplement to 60 mM. Meanwhile, 0.1 mL of samples was taken and added into 0.9 mL of deionized water, which were used for sugar component analysis by HPLC as mentioned above. After about 9 h of reaction, a certain amount of solid sucrose was added into the reaction mixture. Two parallel experiments were carried out.

### Scale-up biotransformation in 5000-L reactor

The scale-up production was performed in the fermentation plant of Zhejiang Meidi Biotechnology Co., Ltd. (Xinchang, Zhejiang, China). A seed culture was prepared in a 5 L flask containing 500 mL of LB medium supplemented with 50 μg/mL of kanamycin at 37 °C, 220 rpm. A second-stage seed cultures were grown in a 50 L fermenter (Eastbio, Zhenjiang, China) containing 25 L of fermentation medium supplemented with 50 μg/mL of kanamycin for 5 h at 37 °C, 300 rpm. Fed-batch fermentation was carried out in a 500 L fermenter (Eastbio, Zhenjiang, China) with 200 L of fermentation medium. The culture conditions including temperature, pH, dissolved oxygen and feeding speed were the same as mentioned above. A cell culture with ~ 100 of OD_600_ was obtained and glycosylation activity was analyzed. All cell culture was transferred into a 5000 L reactor. Sucrose added to 1.2 M and deionized water was added to a certain volume such as 3500 L and 4000 L, and then HQ was constantly added to 50 mM. The batch-feeding whole-cell catalysis was carried out in the 5000-L reactor.

## Results

### Better catalytic performance by recombinant *E. coli* cells than purified amylosucrase

**Fig. 2:**
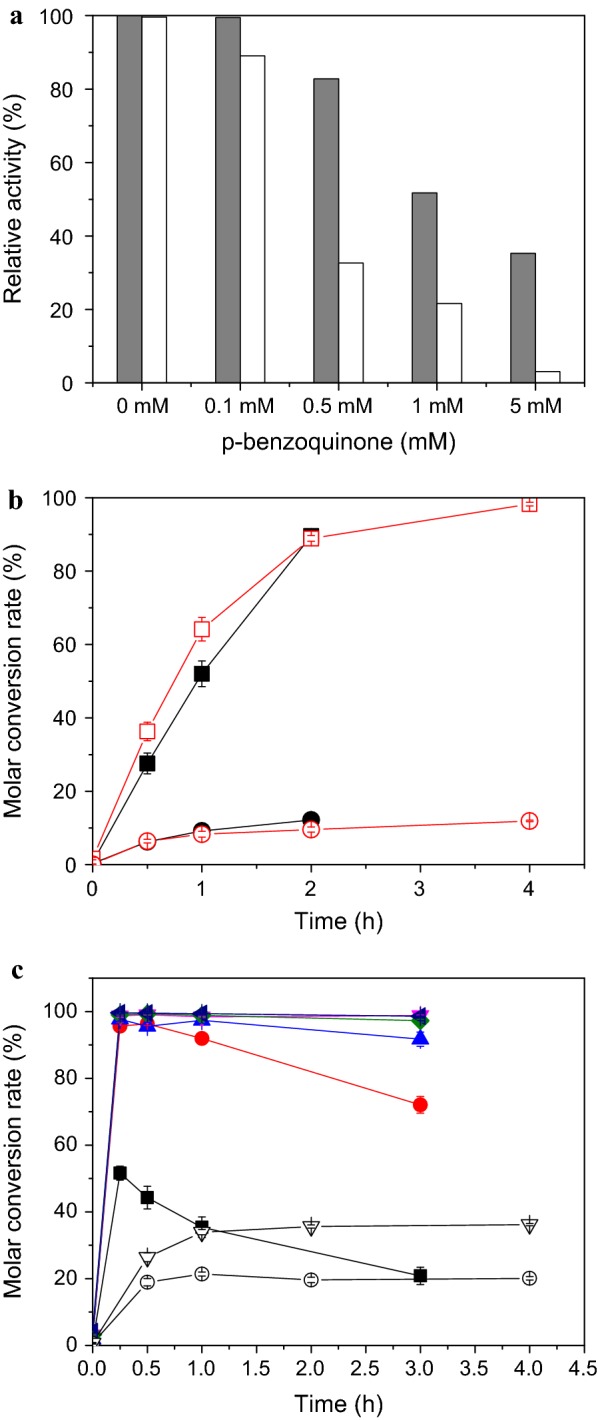
Comparison of catalytic characteristics between purified enzyme (Amy-1) (open) and whole cells of recombinant *E. coli o*verexpressing Amy-1 (close). **a** The tolerance against *p*-benzoquinone of whole-cell catalyst (gray) and purified Amy-1 (white). The reaction mixture contained 20 OD_600_ of cell suspension (1.6 U/mL) or 1.6 U/mL of purified Amy-1, 30 mM of HQ, 1.2 M of sucrose, and different initial concentration of *p*-benzoquinone. **b** The substrate inhibition of hydroquinone on whole-cell catalyst and purified Amy-1. The reaction mixture contained 5 OD_600_ of cell (0.4 U/mL) or 0.4 U/mL of Amy-1, 1.2 M of sucrose, and 20 mM (square) or 80 mM (circle) of hydroquinone in 50 mM PBS (pH 7.0). **c** The effect of different molar ratio of glycoside donor sucrose and glycoside acceptor hydroquinone on whole-cell catalyst and purified Amy-1. Molar ratio: 1:1 (square), 5:1 (circle), 10:1 (up triangle), 15:1 (down triangle), 20:1 (diamond), 40:1 (left triangle). Different molar ratios were prepared by varying the sucrose concentration (30 mM–1.2 M) with constant HQ concentration (30 mM). The reactions were catalyzed by 10 OD_600_ of cell (0.8 U/mL) or 0.8 U/mL of Amy-1 at 30 °C

Amylosucrase Amy-1 from *X. campestris* pv. *campestris* 8004 could glycosylate HQ with high yield and high conversion rate as we previously reported (Zhu et al. 2019) (Fig. 1), but its activity was seriously inhibited by oxidized HQ products. In contrast, whole-cell catalysis displayed a better catalytic performance. For example, whole-cell catalyst behaved a much better tolerance against *p*-benzoquinone as showed in Fig. 2a. Addition of 0.5 mM of *p*-benzoquinone cause only 17% decline in glycosylation rate for whole cells in comparison to 67% decline for the purified enzyme. 10-fold higher glycosylation rate was maintained by whole-cell catalysis when 5 mM of *p*-benzoquinone was added. The substrate HQ also caused serious inhibition, while whole-cell catalyst displayed a similar susceptibility to HQ (Fig. 2b) in comparison with the purified enzyme. Hence, the batch-feeding bioconversion strategy was still required when whole-cell catalyst was used. Interestingly, a very high molar ratio of glycoside donor and acceptor was no longer required for whole-cell catalyst (Fig. 2c), in contrast, as high as 80:1 of sucrose and HQ was required purified Amy-1 to obtain a high conversion rate (> 99%). When whole cells were used for catalysis, 5:1 and 15:1 of molar ratio of sucrose and HQ could achieve > 95% and > 99% of molar conversion rate, respectively. Therefore, catalyst of recombinant *E. coli* cells displayed a much better catalytic performance than purified amylosucrase Amy-1.

### Optimal conditions for α-arbutin synthesis by whole-cell biotransformation

**Fig. 3:**
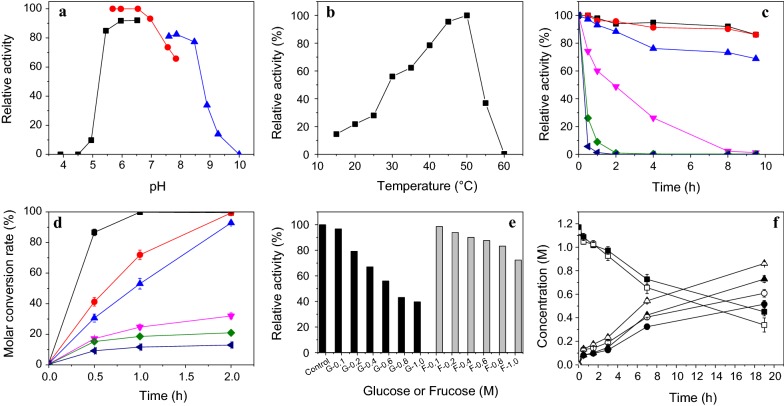
Optimal conditions of whole-cells biotransformation for synthesis of α-arbutin. **a** Optimal pH. 50 mM of sodium acetate buffer (pH 3.9–6.5) (square), 50 mM of PBS (pH 5.6–7.8) (circle), and 10 mM of H_3_BO_3_-NaOH-KCl buffer (pH 7.6–10.0) (up triangle). **b** Optimal temperature. **c** Thermostability at 20 °C (square), 25 °C (circle), 30 °C (up triangle), 35 °C (down triangle), 40 °C (diamond), and 45 °C (left triangle). **d** Different initial concentration of hydroquinone like (mM) 20 (square), 40 (circle), 50 (up triangle), 80 (down triangle), 100 (diamond), 120 (left triangle). **e** The inhibitory effect of different initial concentration of fructose (black) and glucose (gray). **f** Sucrose hydrolysis activity by 5.0 (close) and 10.0 (open) of cell density (OD_600_) without addition of hydroquinone. Square: sucrose, circle: glucose, triangle: fructose

For optimal conditions of whole-cell bioconversion were further evaluated (Fig. 3). In contrast to purified Amy-1, whole-cell catalyst could effectively function in a wider range of pH (5.0–8.5), optimal pH 5.5–6.5 in PBS (Fig. 3a). The fastest glycosylation rate within the first 15 min happened at 50 °C in the range fo 15–60 °C (Fig. 3b), which was 15 °C higher than the purified Amy-1. However, the whole-cell catalyst also displayed poor thermostability and was almost completely inactivated at 45 °C for 1 h (Fig. 3c). It could keep > 68% of initial activity after 9.5 h at 30 °C. Therefore, the optimal catalytic temperature by whole cells was also 30 °C, which was the same as the purified Amy-1.

To avoid the inhibition of substrate, 50–80 mM of initial HQ concentration was recommended for batch-feeding catalysis process (Fig. 3d). Among main products including α-arbutin, fructose and glucose, α-arbutin almost did not cause any inhibition (similar to purified Amy-1), 1.0 M of fructose only caused < 18% inhibition, while 0.6–1.0 M of glucose would cause 46–60% decline in glycosylation rate (Fig. 3e). Glucose was produced due to sucrose hydrolysis by Amy-1 (Fig. 3f). Hence, competitive relations were found between glycosylation and hydrolysis. A high glycosylation activity of whole-cell catalyst might cause excess hydrolysis of sucrose, resulting in high concentration of glucose. For example in Fig. 3f, in comparison to 5 of OD_600_ of cell density (0.4 U/mL), 10 of OD_600_ caused faster accumulation of glucose and up to 0.6 M of glucose was produced after 19 h in absent of HQ.

### High-yield synthesis of α-arbutin by batch-feeding whole-cell catalysis

**Fig. 4:**
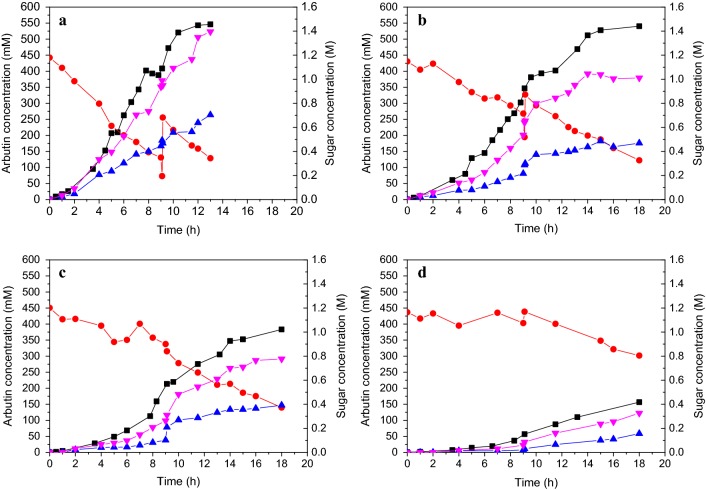
The batch-feeding whole-cell catalysis processes catalyzed by different cell density in a laboratory scale. The 50 mL reaction mixture contained 1.2 M sucrose, 60 mM initial concentration of hydroquinone, 50 mM PBS (pH 7.0), and different cell density including **a** 20.0 of OD_600_ (1.6 U/mL); **b** 10.0 of OD_600_ (0.8 U/mL); **c** 5.0 of OD_600_ (0.4 U/mL); **d** 1.0 of OD_600_ (0.08 U/mL). The reactions were performed in 100 mL round-bottom flask incubated in a thermostatic waterbaths with a magnetic stirrer at 30 °C. Hydroquinone was constantly added into reaction mixture. Sucrose was also supplemented at 9 h in the reaction (**a**, **b**). Square: α-arbutin, circle: sucrose, up triangle: glucose, down triangle: fructose

Batch-feeding strategy was used to avoid substrate inhibition, while the feeding rate of HQ was determined by the glycosylation activity of the whole-cell catalyst, which was also related to sucrose hydrolysis and glucose accumulation. To explore the interrelation between glycosylation rate, HQ-fed rate, and sucrose hydrolysis rate, different cell density was used for batch-feeding whole-cell catalysis and the concentrations of sucrose, α-arbutin, glucose and fructose were monitored (Fig. 4).

According to the changes of α-arbutin concentration, the whole glycosylation process could be divided into three steps including initial step, fast step and balancing step regardless of the cell density. For example, for a high cell density like 20 of OD_600_ (1.6 U/mL) was used (Fig. 4a), Within 13 h of catalysis process, the first 2 h was designated as initial step with 17.4 mM h^−1^ of productivity, 2–10 h was designated as fast step with 55.6 mM h^−1^ of productivity, and 10–13 h was designated as balancing step with 8.7 mM h^−1^ of productivity. The final concentration of α-arbutin was 546 mM. When 10 of OD_600_ (0.8 U/mL) was used (Fig. 4b), the three steps were 0–4 h (17.4 mM h^−1^), 4–14 h (45.4 mM h^−1^) and 14–18 h (7.1 mM h^−1^), respectively, and final concentration was 540 mM of α-arbutin. For 5.0 of OD_600_ (0.4 U/mL) (Fig. 4c), the three steps were 0–6 h (11.4 mM h^−1^), 6–14 h (34.8 mM h^−1^) and 14–18 h (9 mM h^−1^), respectively, and final concentration was 383 mM of α-arbutin. For 1.0 of OD_600_ (0.08 U/mL) (Fig. 4d), only initial step (0–6.6 h) and fast step (6.6–18 h) were observed due to the low enzymatic activity, and final concentration of α-arbutin was 156 mM.

By comparing concentration changes of sucrose, fructose and glucose, transglycosylation reaction and hydrolysis reaction simultaneously happened during the whole catalysis process. Higher glycosylation activity resulted in faster sucrose hydrolysis rate. Therefore, substantial hydrolysis of sucrose occurred and glycosylation rate suddenly declined at 7–9 h of catalysis reaction by 20 of OD_600_. Sucrose was added to restore glycosylation rate when its concentration decreased to below 0.5 M. In addition, sucrose continued to be hydrolyzed even at balancing step. For example, concentrations of fructose and glucose still quickly increased at 10–13 h (Fig. 4a), resulting in the accumulation of glucose at high concentration (up to 0.7 M at13 h) and significant inhibition of glycosylation. The activity of the whole-cell catalyst would decline due to poor thermostability, for example, when reactions were catalyzed by 10 and 5.0 of OD_600_, glycosylation rate and hydrolysis rate both decreased after 14 h of reaction (Fig. 4b, c). Therefore, the reaction catalyzed by 10 of OD_600_ (0.8 U/mL) was optimal for high-yield production of α-arbutin.

### Scale-up production of α-arbutin in 5000 L reactor

**Fig. 5:**
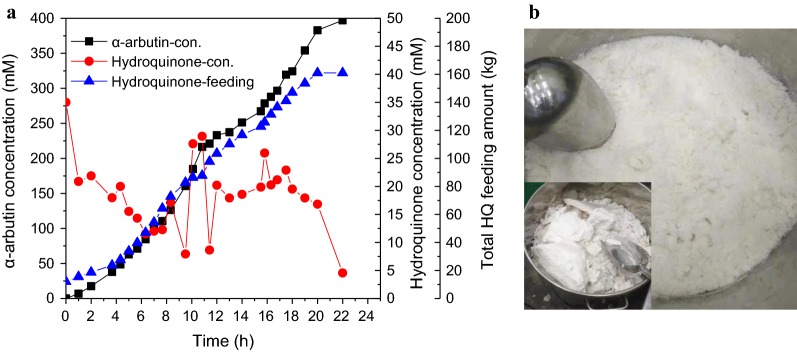
Batch-feeding whole-cell biotransformation for industrial-scale production of α-arbutin in a 5000-L reactor. **a** The concentration of α-arbutin and hydroquinone in reaction mixture and the accumulated amount of hydroquinone during the catalysis process of Batch 1#. **b** The picture of purified product of α-arbutin. Square: the concentration of α-arbutin, circle: the concentration of hydroquinone, triangle: total feeding amount of hydroquinone

The production of α-arbutin by batch-feeding whole-cell biotransformation was scaled up in 5000-L reactor. ~ 10.0 of OD_600_ of whole-cell catalyst was used. Two batches were examined as showed in Table 1 and Fig. 5a (Batch 1#). The total volume in Batch 1# and Batch 2# was 3500 L and 4000 L, respectively. According to the synthetic curve of α-arbutin shown in Fig. 5a, the first 3.5 h was designated as initial step with 10.3 mM h^−1^ of productivity, 3.5–20 h as fast step with 21.2 mM h^−1^ and 20–22 h as balancing step with 7.2 mM h^−1^. A significant decline of glycosylation rate happened at about 11 h during the fast step, and sucrose was then added to restore the glycosylation rate. HQ concentration was continuously examined by HPLC, and supplemented to 50 mM after each analysis. The final concentration of α-arbutin in Batch 1# and Batch 2# was 397 mM and 375 mM and molar conversion rate was 95% and 94.2%, respectively. Comparing to the catalysis in the laboratory scale (Fig. 4b), the final yield decreased by 36–44%, which might result from the declined glycosylation activity of whole-cell catalyst prepared in a 500-L fermenter. Whereas, the molar conversion rate was almost equivalent to that in laboratory scale. The yield was still in the highest level by comparing to previous report (Yang et al. 2019; Zhu et al. 2018). Therefore, the scale-up production of α-arbutin by batch-feeding whole-cell biotransformation is satisfactory. In addition, the product of α-arbutin could be rapidly separated from whole-cell catalyst by microfiltration equipment, and refined to highly-purified product as showed in Fig. 5b (purity > 99%).

**Table 1:** Related parameters in the scale-up biotransformation process of 5000-L reactor

Parameters	Batch 1#	Batch 2#
Final concentration, mM		
α-Arbutin	397	375
Hydroquinone	4.54	6.18
Total yield of α-arbutin, kg	~ 378	~ 408
Total feeding amount of Hydroquinone, kg	161	175
Total amount of sucrose, kg	1150	1600
Final volume, kL	~ 3.5	~ 4.0
Catalytic time, h	22	22.5
Productivity, kg·kL^−1^ h^−1^	4.91	4.53
Molar conversion rate, %	95%	94.2%

## Discussion

Environment-friendly one-step enzymatic transformation for synthesis of α-arbutin attracted wide interests due to rapid growth in global skin-whitening cosmetics market (Migas and Krauze-Baranowska 2015; Zhu et al. 2018). We recently identified the amylosucrase (Amy-1) from *X. campestris* pv. *campestris* 8004 as a new HQ glycosylation enzyme with the highest activity by comparing to other enzymes (Zhu et al. 2018, 2019). In this study, whole-cell catalysis was proved to remarkably outperform enzymatic catalysis including better tolerance to oxidation products of HQ, lower ratio of glycoside donor and acceptor, no decrease in conversion rate. Therefore, a higher concentration (546 mM, 148.5 g/L) of α-arbutin was obtained by whole-cell catalysis than that by purified enzyme as previously reported (Zhu et al. 2019). In addition, whole-cell catalysis has inherent advantages like no need of cell lysis and enzyme purification and easy-to-scale-up of process (Wachtmeister and Rother 2016). In this study, production process was scaled up in 5000 L reactor. Cell cultures were prepared in 500 L fermenter by high-density fermentation, and then were diluted by 10-fold for direct use as catalyst. Its activity declined by 30–40% due to the lack of kanamycin in comparison with the cells cultured in 2.5 L fermenter. The final concentrations of 375–397 mM (102–108 g/L), molar conversion rate (~ 95%) and productivity (4.53–4.91 kg kL^−1^ h^−1^) were satisfactory, which were much higher than those of scale-up production by the cells of *Xanthomonas* BT-112 (Wei et al. 2016) and recombinant *E. coli* cells (Yang et al. 2019). Therefore, batch-feeding whole-cell biotransformation by recombinant *E. coli* cells expressing Amy-1 is effective for industrial production of α-arbutin.

According to the optimal conditions for whole-cell catalysis, cell microenvironment enhanced the tolerance against *p*-benzoquinone and broadened pH range, but did not provided a protective function on the thermostability and susceptibility to HQ. It is still essential to use feeding strategy by constantly monitoring HQ concentration for whole-cell catalysis. Although a lower mole ratio of glycoside donor and acceptor was required by whole-cell catalyst, a very high initial concentration of sucrose (1.2 M) was still used. This is because of the fact that a high concentration of sucrose can decrease sucrose hydrolysis rate, which is similar with other glycosylation reactions catalyzed by glycosidase (Kurosu et al. 2002).

Amylosucrase is a sucrose-utilizing multifunctional enzyme. It can catalyze a variety of tranglycosylation reactions (polymerization, isomerization and glycosylation) and hydrolysis reaction (Tian et al. 2018). Only four main sugar-related compounds were detected in the reaction mixture including sucrose, glucose, fructose and α-arbutin, meaning HQ glycosylation reaction and sucrose hydrolysis reaction were dominant in the catalytic process. Factors influencing these two reactions included HQ-fed rate, catalyst’s activity, sucrose concertation, and glucose accumulation. HQ-fed rate need to be carefully controlled in order to avoid HQ inhibition and excess hydrolysis of sucrose. An excess loading of catalyst (like 20 of OD_600_ in Fig. 4a) would result in a substantial hydrolysis of sucrose and with glucose accumulation, which inhibits HQ glycosylation. Sucrose need to be supplemented if its concentration declined to < 0.5 M during fast step of catalysis process. Therefore, it is important to balance glycosylation rate and hydrolysis rate by controlling these operational parameters for high-yield production of α-arbutin.

Overall, our results proved that batch-feeding whole-cell biotransformation was easy to be scaled up for industrial production of α-arbutin. Scale-up of catalysis process should carefully adjust HQ-fed rate. The product of α-arbutin can be easily separated from whole-cell catalyst by microfiltration equipment, besides, the high product yield and conversion rate also can cut down the cost of the downstream process for α-arbutin refining. Besides, considering the employment of sucrose with low cost, the one-step biotransformation can be successfully applied for industrial production of α-arbutin.

## Data Availability

The datasets supporting the conclusions of this article are included within the article.

## Abbreviations

HQ: hydroquinone
LB: Luria–Bertani
PBS: phosphate buffer system
OD_600_: optical density at 600 nm
